# RETRACTED: Al-Wahaibi et al. Optimized Conjugation of Fluvastatin to HIV-1 TAT Displays Enhanced Pro-Apoptotic Activity in HepG2 Cells. *Int. J. Mol. Sci.* 2020, *21*, 4138

**DOI:** 10.3390/ijms252212012

**Published:** 2024-11-08

**Authors:** Lamya H. Al-Wahaibi, Muneera S. M. Al-Saleem, Osama A. A. Ahmed, Usama A. Fahmy, Nabil A. Alhakamy, Basma G. Eid, Ashraf B. Abdel-Naim, Wael M. Abdel-Mageed, Maha M. AlRasheed, Gamal A. Shazly

**Affiliations:** 1 Department of Chemistry, Science College, Princess Nourah Bint Abdulrahman University, Riyadh 11671, Saudi Arabia; lhalwahaibi@pnu.edu.sa (L.H.A.-W.); msalsaleem@pnu.edu.sa (M.S.M.A.-S.); 2 Department of Pharmaceutics, Faculty of Pharmacy, King Abdulaziz University, Jeddah 21589, Saudi Arabia; uahmedkauedu.sa@kau.edu.sa (U.A.F.); nalhakamy@kau.edu.sa (N.A.A.); 3 Department of Pharmacology and Toxicology, Faculty of Pharmacy, King Abdulaziz University, Jeddah 21589, Saudi Arabia; beid@kau.edu.sa (B.G.E.); abnaim@yahoo.com (A.B.A.-N.); 4 Department of Pharmacognosy, College of Pharmacy, King Saud University, P.O. Box 2457, Riyadh 11451, Saudi Arabia; wabdelmageed@ksu.edu.sa; 5 Department of Clinical Pharmacy, College of Pharmacy, King Saud University, P.O. Box 2457, Riyadh 11451, Saudi Arabia; mahalrasheed@ksu.edu.sa; 6 Department of Pharmaceutics, College of Pharmacy, King Saud University, P.O. Box 2457, Riyadh 11451, Saudi Arabia; gaahmed@ksu.edu.sa

The journal retracts the article titled “Optimized Conjugation of Fluvastatin to HIV-1 TAT Displays Enhanced Pro-Apoptotic Activity in HepG2 Cells” [1], cited above.

Following publication, concerns were brought to the attention of the Editorial Office regarding inappropriate image modification and duplication between this publication [1] and an earlier article [2] published by the same author group.

Adhering to our standard procedure, the Editorial Office and Editorial Board conducted an investigation that confirmed image modification and duplication between Figure 5A,B [1], and Figure 7A,B [2]. Following discussions, the authors were unable to satisfactorily explain these images, nor provide appropriate raw material for Editorial Board evaluation. Consequently, the Editorial Board has lost confidence in the reliability of the findings and decided to retract this publication [1], as per MDPI’s retraction policy (https://www.mdpi.com/ethics#_bookmark30).

This retraction was approved by the Editor-in Chief of the *Int. J. Mol. Sci*.

The authors did not agree to this retraction.
